# Medicinal cannabis for symptom control in advanced cancer: a double-blind, placebo-controlled, randomised clinical trial of 1:1 tetrahydrocannabinol and cannabidiol

**DOI:** 10.1007/s00520-025-09763-5

**Published:** 2025-07-24

**Authors:** Janet R. Hardy, Ristan M. Greer, Anita M. Pelecanos, Georgie E. Huggett, Alison M. Kearney, Taylan H. Gurgenci, Phillip D. Good

**Affiliations:** 1 Mater Research Institute, University of Queensland, Aubigny Place, Mater Health SEQ, Brisbane, QLD 4101 Australia; 2 Torus Research, Brisbane, Australia; 3 Statistics Unit, QIMR Berghofer Medical Research Institute, Herston, Brisbane, Australia; 4 School of Nursing, Midwifery and Social Work, University of Queensland, Brisbane, Australia; 5 Department of Palliative and Supportive Care, Mater Misericordiae Ltd, South East Queensland, Brisbane, Australia; 6 Department of Palliative and Supportive Care Service, Royal Brisbane and Women’s Hospital, Herston, Brisbane, QLD Australia; 7 Faculty of Medicine, The University of Queensland, Herston, Brisbane, QLD Australia; 8 Department of Palliative Care, St Vincent’s Private Hospital Brisbane, Brisbane, QLD Australia

**Keywords:** Medicinal cannabis, Advanced cancer, Palliative care, Symptom control

## Abstract

**Purpose:**

Patients with cancer commonly access cannabis hoping to relieve their symptoms. This study assessed whether a 1:1 10 mg/ml THC:CBD combination oil could improve total symptom burden in patients with advanced cancer over that provided by palliative care alone.

**Methods:**

Participants were randomised to medicinal cannabis (MC) or placebo oil; dose escalated over 14 days according to tolerance and efficacy and continued to day 28. Symptoms assessed using the Edmonton Symptom Assessment Scale (ESAS) were summated to give a total symptom distress score (TSDS). The primary outcome measure was the change from baseline in TSDS at day 14. Secondary outcomes included individual symptom scores, opioid use, participant-selected dose, QoL, psychological symptoms, global impression of change (GIC), and adverse effects.

**Results:**

The pre-planned sample size of 120 at day 14 was reached following the randomisation of 144 patients. Mean (SD) TSDS improved over time in both arms (− 6.30 (12.3) MC, − 6.98 (12.56) placebo, *p* = 0.76) to day 14 with no difference between arms. A statistically significant improvement in ESAS pain scores in the MC arm (mean (SD) − 1.42 (2.15) MC and − 0.46 (2.83) placebo, *p* = 0.04) was at the expense of greater psychomimetic toxicity. Improvement in general well-being was greater for the placebo. GIC and the pain component of QoL both favoured MC.

**Conclusions:**

Patients can be informed that a 1:1 THC:CBD combination cannabis oil was no better than palliative care alone in palliating symptoms in patients with advanced cancer. A small benefit in pain control was associated with greater toxicity.

**Trial registration:**

Australian New Zealand Clinical Trial Registry (ANZCTR): ACTRN12619000037101, 14/01/2019.

**Supplementary Information:**

The online version contains supplementary material available at 10.1007/s00520-025-09763-5.

## Introduction

Cannabis is commonly used by people with cancer for the relief of symptoms related to both the disease and its treatment [1, 2]. Medicinal cannabis has been legalised in many countries for a range of conditions including for “palliative care” despite a paucity of quality evidence to support its use. Much research has been undertaken to establish a therapeutic role for cannabis and multiple systematic reviews have been published [3, 4]. Reviews that include uncontrolled studies, cohort studies, and case reports generally report benefit for several indications [5], whereas reviews that include only controlled studies report no, or only minimal benefit [6, 7]. Despite the lack of data and the increased number of adverse effects in cannabinoid arms compared to placebo, some guidelines and reviews support the prescription of cannabinoids for “palliative care” for refractory situations or when no other options are available [4, 8]. Specialty society guidelines point to the lack of evidence that would allow this recommendation [9–12].

Therefore, we have undertaken a series of studies to determine the role, if any, of cannabinoids in patients receiving palliative care. Rather than focusing on individual symptoms, we have chosen to study total symptom burden to determine why cannabis remains so popular amongst cancer patients. This holistic approach aimed to capture the improvement in general well-being reported anecdotally by many who have used cannabis.

Our randomised trial of synthetic cannabidiol (CBD) (MedCan1) failed to show benefit over placebo in reducing total symptom burden [13]. Tetrahydrocannabinol (THC) is psychoactive and is purported to have a range of benefits including analgesia, anti-nausea, and muscle relaxation [14]. THC is popular amongst cannabis users [15] and is often used in conjunction with CBD in an attempt to ameliorate adverse effects [16]. Furthermore, some have proposed a synergistic effect of different cannabinoids acting at different receptors [17]. This second trial in our program aimed to determine whether the combination of THC and CBD was superior to placebo for symptom control.

## Methods

In this multicenter, randomised, placebo-controlled, double-blind, two-arm parallel trial of an oral 1:1 THC and CBD combination oil versus placebo, doses were titrated upwards as tolerated by participants over a 2-week dose selection phase followed by a 2-week data collection phase. The protocol has been published in full [18].

The trial was sponsored by Mater Misericordiae Ltd with approval from the Mater Human Research Ethics Committee (HREC/MML/49348). An independent data safety monitoring committee (DSMC) oversaw the running of the trial. All participants provided fully informed written consent.

### Participants

Participants were recruited from in-patient, out-patient, and community settings in five metropolitan sites across southeast Queensland. Eligible patients were receiving palliative care for advanced cancer (metastatic or locally advanced) and had an Edmonton Symptom Assessment Scale (ESAS) [19] total symptom distress score (TSDS) of ≥ 10/90 and at least one individual symptom score ≥ 3/10. They had a performance status (Australia-modified Karnofsky Scale (AKPS)) [20] score of ≥ 30/100, a negative THC urine test, could tolerate oral medications, agreed to use no other cannabis products for the duration of the trial, and understood that it is illegal to drive whilst taking THC containing products. Exclusion criteria have been published [18]. In brief, patients were excluded if they had unstable cardiovascular disease, severe hepatic or renal impairment, a history of significant psychiatric disorder, cognitive impairment [21], a known substance use disorder [22], or a history suggesting the risk of drug diversion.

### Random assignment

Full details of random assignment according to Cochrane guidelines are provided in Supplementary Material 1. Randomisation schedules were developed using permuted blocks with randomly allocated block sizes, computer-generated at an independent centre with treatment allocation for each participant placed in opaque envelopes. Stratification was for site only. Participants were randomised in the trial pharmacy according to the set schedule and dispensed active or inactive study drug. All participants, caregivers, investigators, and clinical staff remained blind to study assignment until completion of the data analysis. Study drug and placebo were supplied in an oil solution identical in appearance and matched for taste, colour, and bottle size.

### Study procedure

The cannabis product was a plant-based 1:1 THC/CBD 10 mg/10 mg/ml oral solution supplied by one company and quality-controlled according to Australian regulations. Participants took daily doses of study oil or placebo over the range 0.25–3 ml/day (equivalent to 2.5 mg/2.5–30 mg/30 mg/day THC/CBD for those in the active arm) according to a set dosing schedule (Supp Table 1). Doses could be escalated or reduced according to tolerability and/or perceived efficacy following discussion with research staff.

All participants were given standard palliative care [23] according to the local practice of the recruiting centre. They continued all medications including opioids, antiemetics, sedatives, and specific anti-cancer therapy according to clinical need with dose changes allowed as clinically indicated. Participants received regular phone calls (every 3 days for the first 2 weeks) and out-patient clinic medical review visits at days 7, 14, 21, and 28. Post-COVID, most day 7, 21, and follow-up assessments were done via telehealth.

ESAS [19] is a validated patient-centred tool to measure symptom distress in nine domains: pain, tiredness, nausea, shortness of breath, drowsiness, appetite, anxiety, depression, and feeling of well-being. Each symptom domain is scored from 0 (least severe) to 10 (most severe). The TSDS, the sum of individual ESAS scores, ranges from 0 to 90, measures total symptom burden and is used in both clinical and research settings [24, 25].

Demographic data and study assessments including ESAS scores, opioid use [26], AKPS [20], quality of life (QoL) [27] and Depression Anxiety and Stress Scores (DASS) [28] were collected as per the published protocol [18]. Patient-reported ESAS scores, self-selected doses and Global Impression of Change (GIC) [29] were recorded weekly. Cannabis specific adverse events (CTCAE) V4.03 [30] were assessed weekly and at all telephone reviews. Other adverse events were reported if worse than baseline at any time during the trial and classified according to CTCAE categories.

### Outcome measures

The primary outcome measure, the change from baseline TSDS at day 14, was based on previous work showing a change in TSDS of 5.7 to be the minimal clinically important difference [31]. Secondary outcome measures included the patient-determined effective dose of study drug; TSDS at days 7, 21, and 28; physical and emotional ESAS sub-scores; individual ESAS symptom scores; opioid use; GIC; baseline and change of DASS scores; QoL; survival; and adverse events. Participants who chose to withdraw from the study or whose performance status rendered them unable to continue provided no further data after the date of exit.

### Sample size and analysis

The sample size was based on the minimal clinically important difference in TSDS of 5.7 [31]. Allowing 20% for attrition, and with a predicted improvement of ≥ 6 for the MC arm compared to placebo at day 14, it was planned to randomise 144 participants (72 per treatment arm) to achieve a sample size of 60 participants per arm, assuming 80% power, a Type 1 error of 5% (two-tailed), and a standard deviation of 11.6.

Normally distributed variables are described as mean (standard deviation, SD) or, for hypothesis tests, mean (SE), non-normally distributed variables as median (interquartile range, IQR), unless otherwise specified, and categorical variables as number and percent. Difference from baseline scores (difference scores) were calculated as the value at day 14 (or day 28) minus the baseline value. Normally distributed difference scores were evaluated using *t*-tests, as were normally distributed outcomes evaluated at a single time point. Difference scores adjusted for baseline value using ordinary least squares regression (OLS) [32] were also reported. All data were analysed with subjects assigned to the group to which they were randomised, as per intention to treat (ITT) analysis [33]. The effect of centre was assessed where appropriate.

Non-normally distributed variables were compared using Wilcoxon’s Rank Sum test and categorical variables by Pearson’s chi-square or Fisher’s exact test. Correlations were assessed using Spearman’s rho. For longitudinal data where outcomes were measured at two or more timepoints, generalised estimating equations (GEE) were used to compare the trajectory of response over time, accounting for missing data and repeated measures within subject. The distribution of the outcome variable was assessed and various correlation structures were compared, with the most appropriate correlation structure selected. Time was treated as a continuous rather than categorical variable (except for the QoL analysis). Time in the study to 28 days and overall survival were compared using Kaplan–Meier analysis with the log-rank test.

Anticipating a high attrition rate, sensitivity analyses were carried out to compare results with the pre-specified protocol analyses. These included *t*-tests, GEE, and linear mixed models with and without last observation carried forward (LOCF) data imputation [34]. Participant characteristics at baseline and at day 14 were compared to assess any imbalance in attrition. Significance was pre-defined as *p* ≤ 0.05. No multiple comparison adjustment was performed. Analysis was carried out using R version 4.4.2 [35].

## Results

**Fig. 1:**
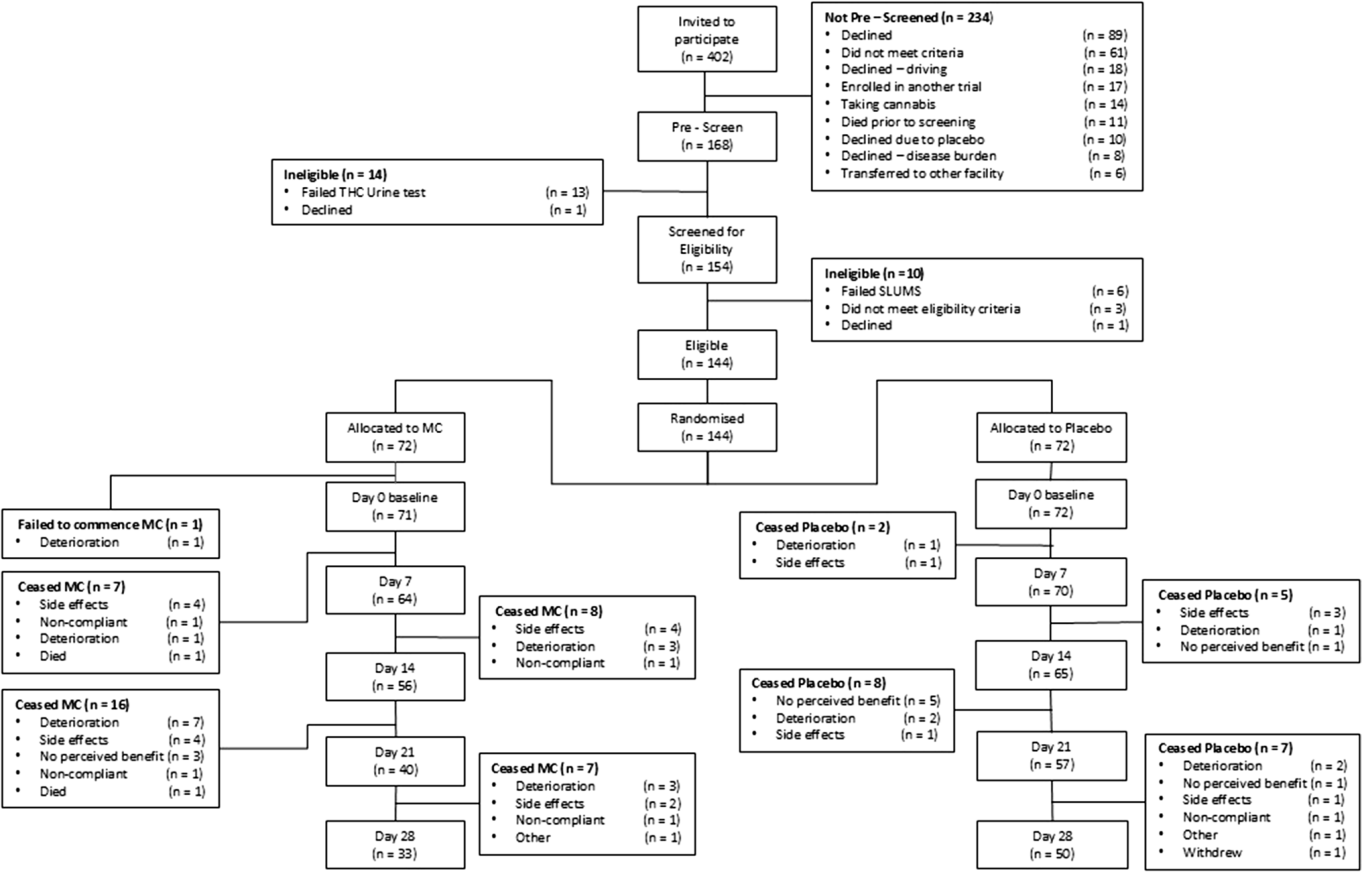
CONSORT diagram. THC, tetrahydrocannabinol; SLUMS, St Louis University Mental Status Examination; MC, medicinal cannabis

From September 2019 to July 2023, 144 patients were randomised to reach the pre-planned sample size of 120 at day 14. Participant flow is shown in Fig. 1. Most participants (115/145 (79%)) were recruited at the primary centre. One participant was randomised but deteriorated and was unable to commence the study. One other patient, thought to have locally advanced cervix cancer but subsequently found to have no active disease, was included in the analysis. The attrition rate was high; 16/72 (22.2%) MC and 7/72 (9.7%) placebo participants exited before day 14 and 39/72 (54.2%) MC and 22/72 (30.5%) placebo before day 28 (p = 0.007). Common reasons for exiting were deteriorating condition (17/72 (23.6%) MC, 6/72(8.3%) placebo), side-effects (14/72 (19.4%) MC, 6/72 (8.3%) placebo), and lack of patient perceived benefit (3/72 (4.1%) MC, 7/72 (9.7%) placebo). There was no difference in the proportion withdrawing due to side effects compared with withdrawal for other reasons (data on request).

Baseline demographics are shown in Table 1. Participants were of reasonable performance status (median AKPS 70%), with a median age of 65 years (IQR 56–73). The most common cancers were gynaecological, breast, and lung. Median (IQR) baseline TSDS was 36.0 (27.5–49.5) for MC and 36.0 (21.8–46.0) for placebo. The median number of symptoms per participant was 8 (range 3–9). The arms were well matched at baseline except for opioid use; 61/71 (85.9%) of participants on MC and 49/72 (68.1%) on placebo were taking opioids. Those on opioids were on a similar OME dose (80 mg/day (IQR 40.0–140.0) MC and 70 mg/day (IQR 30.0–140.0) placebo). Similar numbers in each arm were taking antidepressants, antipsychotics, and benzodiazepines at baseline. Many participants in each arm were receiving anticancer medications (41/71 (58%) MC, 35/72 (49%) placebo), *p* = 0.27, and/or corticosteroids (41/71 (58%) MC, 38/72 (53%) placebo), *p* = 0.55.

**Table 1:** Baseline demographics

Characteristic	THC/CBD ((*n* = 71)	Placebo (*n* = 72)	All participants (*n* = 143)
Age, years, median (range)	65 (36–90)	65.5 (36–88)	65 (36–90)
Sex (male), No. (%)	27 (38.0)	30 (41.7)	57 (39.9)
Primary cancer			
Gynaecological	17 (23.9)	15 (20.8)	32 (22.4)
Lung	14 (19.7)	7 (9.7)	21 (14.7)
Breast	9 (12.7)	12 (16.7)	21 (14.7)
Other GIT	7 (9.9)	5 (6.9)	12 (8.4)
Colorectal	5 (7.0)	7 (9.7)	12 (8.4)
Prostate	2 (2.8)	8 (11.1)	10 (7.0)
Skin	0 (0.0)	5 (6.9)	5 (3.5)
Unknown	1 (1.4)	1 (1.4)	2 (1.4)
Other	16 (22.5)	12 (16.7)	28 (19.6)
ESAS (TSDS), mean (SD)	37.6 (14.7)	36.5 (16.4)	37.0 (15.5)
ESAS (individual symptoms) mean (SD)			
Pain	4.59 (2.42)	4.46 (2.75)	4.52 (2.59)
Nausea	2.86 (3.06)	2.57 (3.01)	2.71 (3.03)
Tiredness	5.79 (2.43)	6.13 (2.22)	5.97 (2.32)
Shortness of breath	3.58 (2.67)	3.37 (2.93)	3.48 (2.80)
Drowsy	4.59 (2.47)	4.36 (2.59)	4.48 (2.53)
Appetite	4.38 (2.89)	4.64 (2.99)	4.51 (2.93)
Anxiety	3.86 (3.03)	3.40 (3.11)	3.63 (3.07)
Depression	2.89 (2.97)	2.68 (2.97)	2.78 (2.96)
Wellness	5.09 (2.51)	4.85 (2.32)	4.97 (2.41)
AKPS, median (range)	70 (40–90)	70 (30–100)	70(30.0–100.0)
SLUMS, mean (SD)	26.8 (2.5)	27.2 (2.5)	27.0 (2.5)
Concomitant medications, No. (%)			
Antidepressant	21 (29.6)	27 (37.5)	48 (33.6)
Antipsychotic	10 (14.1)	7 (9.7)	17 (11.9)
Benzodiazepine	15 (21.1)	20 (27.8)	35 (24.5)
Opioids	61 (85.9)	49 (68.1)	110 (76.9)
Opioid dose			
Total opioids, all participants, mg OME, median (range)^a^	60.0 (0–520)	31.5 (0–2210)	45.0 (0.0–2210.0)
Total opioids, excluding those not taking, mg OME, median (range)^b^	80.0 (2–520) *N* = 61	70.0 (1–2210) *N* = 49	74.1 (1–2210) *N* = 110
EORTC QoL, mean (SD)^c^			
Physical functioning	50.2 (26.7)	54.6 (23.6)	52.4 (25.2)
Emotional Functioning	73.5 (23.4)	73.3 (24.9)	73.4 (24.0)
Overall quality of life	50.2 (23.3)	46.9 (22.6)	48.6 (22.9)
	*N* = 71	*N* = 71	*N* = 142
DASS severity rating, No. (%)			
Depression			
Normal/mild	50 (70.4)	50 (69.4)	100 (69.9)
Moderate	14 (19.7)	13 (18.1)	27 (18.9)
Severe/extremely severe	7 (9.9)	9 (12.5)	16 (11.2)
Anxiety			
Normal/mild	39 (54.9)	38 (52.8)	77 (53.8)
Moderate	17 (23.9)	14 (19.4)	31 (21.7)
Severe/extremely severe	15 (21.1)	20 (27.8)	35 (24.5)
Stress			
Normal/mild	52 (73.2)	55 (76.4)	107 (74.8)
Moderate	11 (15.5)	11 (15.3)	22 (15.4)
Severe/extremely severe	8 (11.3)	6 (8.3)	14 (9.8)

*THC/CBD* tetrahydrocannabinol/cannabidiol, *GIT* gastrointestinal, *ESAS* Edmonton Symptom Assessment System, *AKPS* Australian-modified Karnofsky Performance Status Scale, *SLUMS* St Louis University Mental Status, *OME* oral morphine equivalent, *QoL* quality of life (EORTC QLQ-C15-PAL), *DASS* depression, anxiety, stress scale

^a^Includes breakthrough doses and those not on opioids

^b^Excludes those not on opioids (10 on MC, 23 placebo)

^c^Missing data = 1 in placebo group

### Primary analysis: total symptom burden at day 14

**Fig. 2:**
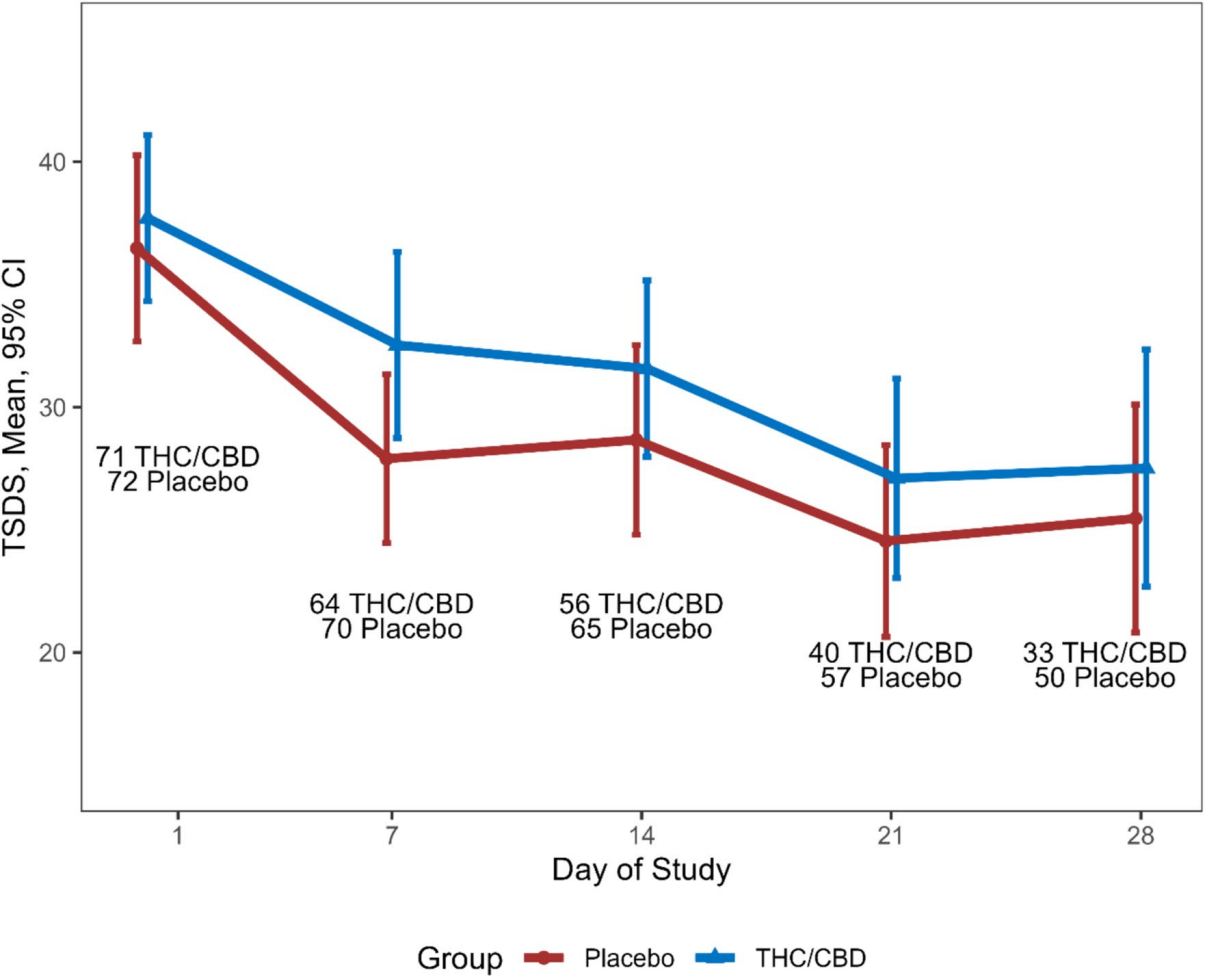
TSDS over time. THC/CBD, tetrahydrocannabinol/ cannabidiol; Pl, placebo; TSDS, total symptom distress score

**Fig. 3:**
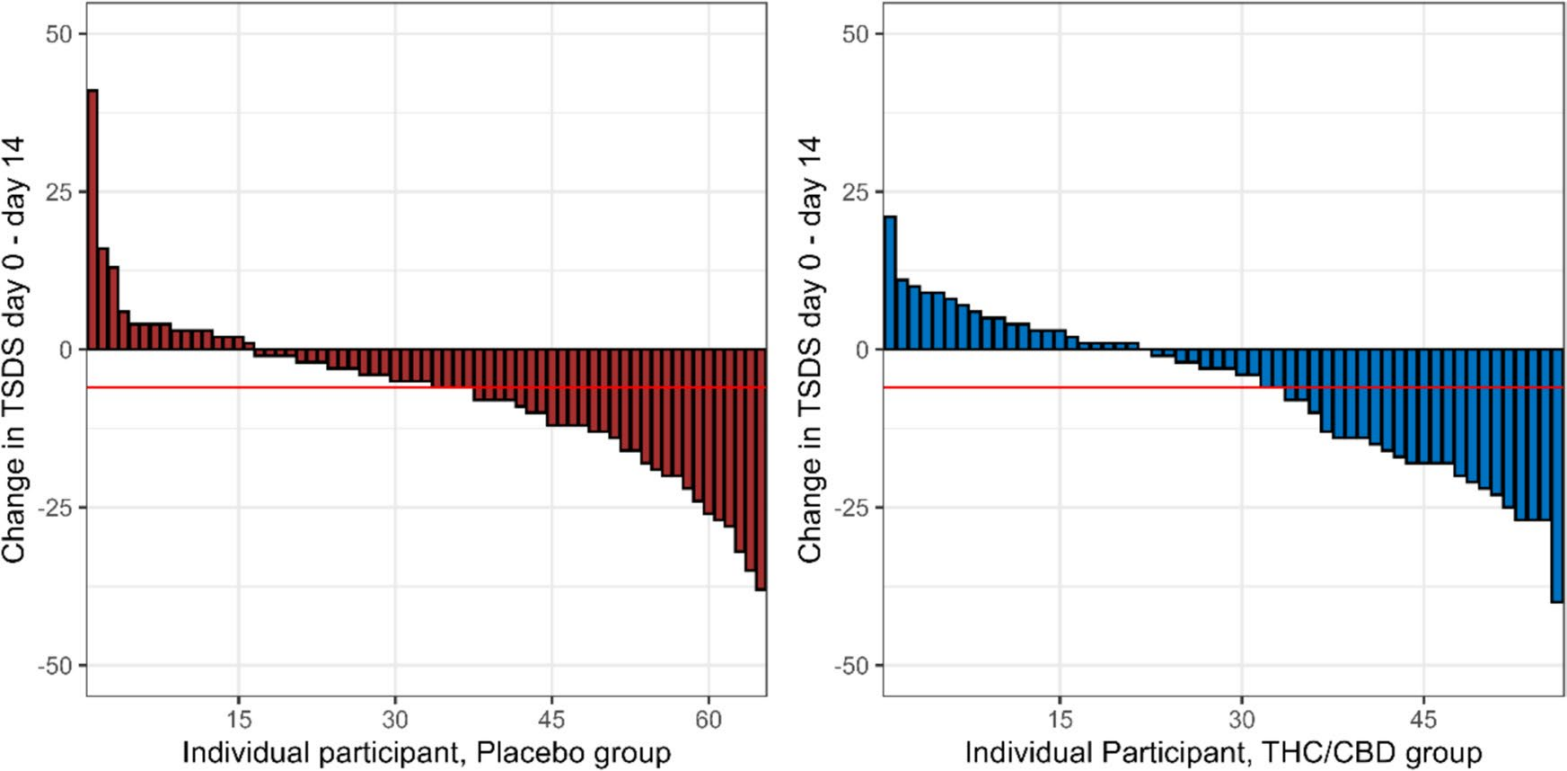
Proportion of responders (fall in TSDS ≥ 6 between baseline and day 14) in each arm. THC/CBD, tetrahydrocannabinol/ cannabidiol;  TSDS, total symptom distress score

Symptom scores improved in both arms over time with a mean (SD) change in TSDS of − 6.30 (12.3) MC and − 6.98 (12.5) for placebo (*p* = 0.76) at day 14 (Fig. 2). Adjusted for baseline, the mean (SE) difference in score change was − 1.45 (2.07), *p* = 0.48. A clinically meaningful reduction in TSDS of ≥ 6 was seen in 32/65 (49.2%) of those on placebo and 25/56 (44.6%) of those on MC, *p* = 0.75 (Fig. 3).

### Secondary analyses

#### Change in TSDS over time (Fig. 2)

There was no difference between arms in change in TSDS from baseline to day 28 (mean (SD) change in TSDS − 9.24 (15.3), *n* = 33, for THC/CBD and − 8.42 (13.6), *n* = 50, for placebo, *p* = 0.80). Adjusted for baseline, the difference in mean (95%CI) change in TSDS was − 0.33 (− 0.47 to 6.13), *p* = 0.91.

#### Individual ESAS symptoms (Table 2)

There was a significant difference in reduction in pain scores (mean (SD) − 1.41 (2.15) for MC and − 0.46 (2.82) placebo) from baseline to day 14 in favour of MC, remaining significant when adjusted for baseline values (mean (SE) − 0.85 (0.42)) (*p* = 0.04). From baseline to day 28, the reduction in pain score was greater in the MC group (mean (SD) − 1.42 (2.29)) than the placebo group (− 0.34 (2.47)), *p* = 0.047. This difference remained when adjusted for baseline (mean (SE) difference in pain score − 1.07 (0.50), *p* = 0.03) Table 2.

**Table 2:** Difference in change in ESAS components by treatment group from baseline to day 14

ESAS component	Change in score THC/CBD, mean (SD) *N* = 56	Change in score placebo, mean (SD) *N* = 65	*p*-value (from *t*-test)	Difference in score change from baseline to day 14, adjusted for baseline value (mean (SE))^b^	*p*-value (from OLS)
Pain	− 1.41 (2.15)	− 0.46 (2.82)	0.04	− 0.85 (0.42)	0.04
Tiredness	− 0.48 (2.83)	− 1.0 (2.63)	0.30	0.39 (0.43)	0.36
Nausea	− 0.34 (3.08)	− 0.71 (2.76)	0.49	0.44 (0.43)	0.31
Shortness of breath	− 0.61 (2.58)	− 0.69 (2.13)	0.84	0.16 (0.40)	0.70
Drowsiness	− 0.14 (2.80)	− 0.40 (2.30)	0.58	0.56 (0.42)	0.19
Appetite	− 0.19 (2.72)	− 0.69 (2.78)	0.32	0.28 (0.43)	0.51
Anxiety	− 1.46 (2.36)	− 1.17 (2.20)	0.48	0.007 (0.33)	0.98
Depression	− 1.17 (2.99)	− 0.57 (2.30)	0.21	− 0.20 (0.38)	0.60
Wellbeing	− 0.48 (2.78)	− 1.29 (2.74)	0.11	0.97 (0.40)	0.02
Physical^a^	− 3.18 (8.31)	− 3.95 (9.43)	0.63	0.96 (1.54)	0.53
Emotional^a^	− 2.64 (4.81)	− 1.73 (4.08)	0.26	− 0.25 (0.65)	0.70

^a^ESAS subcategories

^b^A positive difference in score indicates treatment is in favour of placebo

There was a significant improvement in overall well-being in favour of placebo by day 14 when adjusted for baseline values (0.97 (0.40), *p* = 0.02), not maintained to day 28. There was no difference between arms for any other individual symptom and no difference between physical and emotional ESAS sub-scores.

#### Participant selected dose

Across all participants, the median (IQR) dose of MC was 1.5 ml (0.87–3.0 ml) (corresponding to 15 mg CBD/15 mg THC per day) and 3 ml (2.5–3.0 ml) for placebo (*p* < 0.001).

#### Opioid use

No difference in the proportion of participants increasing, having no change, or decreasing their opioid dose between MC and placebo groups was detected. The median (IQR) opioid dose at each time point was similar between arms as shown (Supp Table 2). There was no correlation between maximum treatment dose of MC and daily OME (Supp Fig. 1). Similarly, there was no significant difference in dose of MC according to benzodiazepine use between arms (data on request).

#### Participant and clinician assessed GIC

There was no difference between treatment groups in the proportion of participants feeling “better” or “much better” at days 7, 14, or 21 compared with baseline. At day 28, a higher proportion of participants in the MC group reported feeling “better” or “much better”, compared with baseline, whether participant (29/33 (88%) MC vs 33/50 (66%) placebo, *p* = 0.05) or clinician (30/33 (91%) vs 31/49 (63%), *p* = 0.01) assessed (Supp Fig. 2).

#### Quality of life

Modelling the trajectory of EORTC domains over time, there was no difference between arms for all EORTC domains except for pain in favour of MC (difference in reduction of pain score/day 0.46 (SE 0.2), *p* = 0.02) (Supp Table 3).

#### Psychological symptoms

Depression, anxiety, and stress all improved slightly over time with no difference between arms (Supp Table 4).

#### Survival

The median survival across all participants was just over 6 months (198 days (95% CI 140–301)) with no difference between arms (Supp Fig. 3).

### Adverse events

Significantly more participants on MC reported confusion, feeling high, and an exaggerated sense of well-being (Table 3) worse than baseline. AEs were generally of low grade (1–2) with few exceptions (one grade ≥ 3 for each of insomnia, nausea, and abnormal liver function (MC) and one grade ≥ 3 for each of abdominal pain and abnormal liver function (placebo)).

**Table 3:** Solicited adverse events at all study time points^a^

Adverse event	THC/CBD	Placebo	*p*-value
Somnolence	34/69 (49.3)	28/72 (38.9)	0.21
Nausea	31/69 (44.9)	24/72 (33.3)	0.16
Concentration	29/69 (42.0)	23/72 (31.9)	0.21
Weakness	28/69 (40.6)	19/72 (26.4)	0.07
**Confusion**	**26/69 (37.7)**	**12/72 (16.7)**	**0.005**
Dizziness	25/69 (36.2)	26/72 (36.1)	0.99
Unsteadiness	22/67 (32.8)	17/71 (23.9)	0.25
Clumsiness	22/68 (32.4)	14/72 (19.4)	0.08
**Feeling high**	**21/69 (29.0)**	**10/72 (13.9)**	**0.02**
Dry mouth	19/69 (27.5)	22/72 (30.6)	0.69
Abdominal pain	19/69 (27.5)	22/72 (30.6)	0.69
Anxiety	17/69 (24.6)	15/72 (20.8)	0.59
Mood change	16/69 (23.2)	16/72 (22.2)	0.89
Vomiting	15/69 (21.8)	10/72 (13.9)	0.22
Coordination	14/68 (20.6)	13/71 (18.3)	0.73
Headache	14/69 (20.3)	18/72 (25.0)	0.50
Diarrhoea	13/69 (18.8)	13/72 (18.1)	0.90
Sweating	12/68 (17.6)	9/70 (12.9)	0.43
Warm/tingling feeling	11/69 (15.9)	8/72 (11.1)	0.40
Insomnia	10/69 (14.5)	14/72 (19.4)	0.43
**Exaggerated sense of well-being**	**10/69 (14.5)**	**2/72 (2.8)**	**0.01**^**b**^
Personality change	9/69 (13.0)	3/72 (4.2)	0.07
Paranoia	2/69 (2.9)	2/72 (2.8)	1.0^b^
Psychosis	1/69 (1.4)	1/72 (1.4)	1.0^b^

Bold items refer to those that are significant i.e. *p* < 0.05

^a^Adverse events reported as new or worse than baseline, study days 2, 4, 7, 9, 11, 14, 21, and 28

^b^Fisher’s exact test

As expected in this population with multiple concomitant medications and anti-cancer treatment, there were many non-solicited AEs with no difference between arms (Supp Table 5) apart from paraesthesia (9 participants on placebo).

During the trial, 13 serious adverse events (SAEs) were reported, most commonly increasing confusion, drowsiness, nausea/vomiting, and/or deteriorating liver function. On unblinding, 10 were in those on MC and 3 on placebo. Thirty non-reportable SAEs (14 placebo, 16 MC) were considered by the DSMC and determined to be directly attributable to underlying disease rather than study drug.

### Sensitivity analyses

Sensitivity analyses for the effect of treatment arm on change in TSDS from baseline to day 14, using imputed data (LOCF) and GEE and mixed models analysis, showed comparable results (data on request).

Any effect on the difference in change in TSDS associated with the imbalance in opioid exposure was examined using an OLS model. Although there was a trend for those on opioids to have less reduction in TSDS, there was little effect on the primary outcome (data on request).

## Discussion

Despite ongoing “real-world” reports supporting the value of medicinal cannabis [4] and the general favourable opinion of the lay public and in the media, it is becoming increasingly difficult to demonstrate medical benefit in controlled trials. There are exceptions in childhood epilepsy [36], spasticity related to multiple sclerosis, inflammatory bowel disease, and possibly in nausea and vomiting, sleep, and chronic pain [4].

In our previous trial, we were unable to show a benefit of synthetic CBD in the symptom management of patients with advanced cancer over that provided by palliative care alone [13]. In this study, the addition of THC to CBD did not affect the primary outcome (total symptom burden) but resulted in a statistically significant improvement of pain. A positive result in favour of MC in the pain component of our QoL assessment supports this finding. The study was not powered for the secondary outcomes, however, and the improvement in pain at 0.85 units is of doubtful clinical significance. An improvement of 1–2 units on an 11-point numerical rating scale is generally considered to be clinically meaningful [24, 37]. This result is consistent with recent meta-analyses that point to a small pain benefit in chronic pain of mixed aetiology [7, 38] and contrasts with reviews that include uncontrolled and observational studies that report wide-ranging benefits, including improvements in pain [5]. Our study is one of very few controlled trials to show a benefit in cancer pain. A Cochrane review of cannabis for adults with cancer pain reports moderate certainty evidence that nabiximols (THC/CBD combination) and THC are ineffective in relieving moderate to severe opioid-refractory cancer pain [6]. Consistent with other RCTs [39, 40], and in contrast with observational studies, we did not demonstrate an opioid sparing effect.

The median dose of 15 mg/day was selected by active arm participants according to perceived efficacy and or dose-limiting toxicity. By day 7, 15 participants on placebo and 42 on MC had self-selected a dose. This supports our study design of starting low and dose escalating according to individual patient response and tolerance.

It has been stated that equal doses of THC/CBD perform better as compared with THC- or CBD-dominant products [41], but this combination is not without toxicity. As might be expected, there was a higher incidence of psychoactive “adverse effects” in the cannabis arm. The adverse effects of MC are seldom reported in the community, but many reviews highlight the potential for adverse effects [4, 38, 39]. As all participants in this study were followed closely by phone and/or in person whilst on trial, many adverse effects were detected early and often adjusted for by dose reduction.

Improvement, as measured by GIC (proportion of participants reporting feeling “better” or “much better” when taking oil), improved over time in both arms. At day 28, significantly more participants in the active arm reported an improvement when both self- and clinician-assessed. This finding is likely to be strongly influenced by those patients who dropped out early because of toxicity or a perceived lack of benefit, however. Moreover, there was a greater improvement in the overall wellbeing item in ESAS in favour of the placebo by day 14.

A potential limitation of this study is that most participants were recruited at the primary centre. Sensitivity analysis adjusting for site found no significant impact on the results, however.

The supply of free drug was advantageous with respect to budget costs but exposed investigators to the perception of bias. The supplier had no role in the development, undertaking, analysis, or reporting of this study and we include a full disclosure of steps undertaken to minimise bias (Supp Material 1).

High attrition rates and missing data are inevitable consequences of undertaking studies in a poor-prognosis population such as this. As no participant provided any further trial data after the date of exit, we utilised a complete-cases intention-to-treat analysis for our primary outcome measure. All sensitivity analyses including data imputation showed similar results to the primary analysis (data on request).

There was greater attrition in the MC arm of the study, but the proportion of those withdrawing from the study for each individual reason did not differ between arms.

Apart from opioid use, there were no statistically significant differences in participant characteristics between the MC and placebo groups at baseline or the primary outcome point.

The higher proportion of participants on opioids may have contributed to the adverse events in the MC arm. In both arms, opioids limited the reduction in TSDS over time, but sensitivity analysis showed this to have little impact on the primary outcome. Moreover, the dose of opioids taken was similar in both arms and the excess of psychomimetic side-effects in the MC arm (feeling high and an exaggerated sense of well-being) would be more consistent with cannabinoids than opioids.

The delivery of cannabis in different formulations and routes (for example, edibles or inhalation) may lead to a greater pain response. Similarly, the proportion of different cannabinoids best suited to a pain response may be important; this is the subject of our subsequent trial [42].

In summary, THC/CBD oil (10 mg/10 mg per ml) led to a small benefit in pain but with increased toxicity and did not reduce overall symptom burden overall in patients with advanced cancer. The improvement in symptom burden over time in both arms could reflect good palliative care or a placebo effect, the impact of which has been clearly demonstrated in other studies [43]. The added benefit to participants of regular follow-up telephone calls cannot be underestimated [44]. The findings of this study are consistent with current literature and should help clinicians when counselling patients on the use of cannabinoids for symptom control.

The trial was registered with the Australian New Zealand Clinical Trial Registry (ACTRN12619000037101p).

## Supplementary Information

Below is the link to the electronic supplementary material.

Supp Material 1 (DOCX 18.7 KB)

Supp Table 1 (DOCX 20.7 KB)

Supp Table 2 (DOCX 21.4 KB)

Supp Table 3 (DOCX 22.9 KB)

Supp Table 4 (DOCX 21.1 KB)

Supp Table 5 (DOCX 29.4 KB)

Supp Figure 1. MC vs OME Scatter plot of maximum dose of oil vs total opioids (OME) at day 14 (PDF 16.4 KB)

Supp Figure 2. Global Impression of Change over time. a) patient assessed b) clinician assessed. THC/CBD, tetrahydrocannabinol/ cannabidiol (PPTX 694 KB)

Supp Figure 3. Survival over time. THC/CBD, tetrahydrocannabinol/ cannabidiol (PDF 8.42 KB)

## Data Availability

Data is provided within the manuscript or supplementary information files. As specified in the text, additional data can be requested from the corresponding author.
