# Supplementary material for: Medicinal cannabis for symptom control in advanced cancer: a double-blind, placebo-controlled, randomised clinical trial of 1:1 tetrahydrocannabinol and cannabidiol
Source: Support Care Cancer. 2025 Jul 24;33(8):715. doi: 10.1007/s00520-025-09763-5 (PMC12289739; doi:10.1007/s00520-025-09763-5)
Supplement: Supplementary file 1 — Supp Material 1 (DOCX 18.7 KB) [file 520_2025_9763_MOESM1_ESM.docx]

Supplementary material 1

Risk of bias (reported according to RoB2: a revised tool for assessing risk of bias in randomised trials. Sterne J et al, BMJ 2019;366:14898)

1. Randomisation process.

Randomisation schedules were developed for each site using random number tables, computer generated at an independent centre (Queensland Institute of Medical Research) by a statistician who played no other role in the study. Treatment for each patient was allocated according to a block randomisation schedule held by the central registry and not disclosed to investigators. Each of the 5 sites had a separate randomisation schedule.

The randomisation schedules were sent to an independent clinical centre where a research assistant with no other role in the study placed notification of the site, participation number and drug allocation (drug 1 or drug 2) in opaque sealed envelopes. The front of the envelop listed the trial site name, and randomisation number only.

The allocation sequence was sent to trial pharmacists in 4 of the trial sites via password protected email. Sites 1 and 2 utilised the same trial pharmacy. Pharmacy randomised the participant according to the schedule and dispensed active or inactive medication in a participant ID labelled bottle that did not indicate the arm.

Two investigators at external sites not directly involved with participant care or randomisation were sent the randomisation schedules under password protected emails in case of the need for emergency unblinding but was not required at any stage throughout the study.

All participants, caregivers, investigators and clinical staff remained blind to study assignment until trial completion and completion of data analysis. Treatment allocation was not disclosed to study staff, treating clinicians or investigators.

All study drugs and placebo were in oil solution form of identical appearance and matched for taste, colour and bottle size to preserve the blinding irrespective of the contents. Each participant received the oil solution in a prepacked bottle labelled with their individual trial participation ID number, and consecutively numbered according to the randomisation scale.

Baseline demographics supported the randomisation process.

2. Deviations from intended interventions.

All participants, relatives, carers, research staff and investigators remained blind to the treatment allocation until all participants had been recruited and data checking and locking processes completed, and data analysis undertaken.

As anticipated, there was a high attrition rate in this study. Reasons for drop out were carefully monitored and are presented. General estimating equations at day 28 were consistent with the primary outcome measure at day 14.

3. Missing data.

As anticipated in a population of patients with advanced disease, there was attrition throughout the study. As no participant provided any further trial data after the date of exit, we utilised a complete-cases intention to treat analysis for our primary outcome measure. A number of sensitivity analyses were undertaken to account for missing data including imputed data (LOCF) and GEE and mixed models analysis. These all showed similar results to the primary analysis. Data for the per protocol analysis of primary and secondary outcomes were available for nearly all participants randomised. One patient randomised was excluded from the trail and analysis as they became too unwell to participate. Data from one patient was missing from the QoL assessment.

4. Measurement of the outcomes

All participants, relatives, carers, research staff and investigators remained blind to the treatment allocation during all trial assessments at all time points. The pre-planned outcome measures were used consistently throughout as per the original protocol.

5. Selection of the reported result.

Results were reported as specified in the original protocol with the exception of:

a). RUG-ADL, Resource Utilization Groups – Activities of Daily Living Scale scores. These were reported at baseline (no difference between arms) but subsequently removed as not discussed at any point in the manuscript and of little relevance.

b). C-reactive protein (CRP). This was initially included as a measure of anti-inflammation over time. CRP levels were very low at baseline in one arm however, rendering the interpretation of results meaningless.

c). Clinician-administered dissociated states scale (CADSS). The intention was to complete this scale if the participant scored >0 on the psychoactive components of the adverse-events assessments. This was not done consistently and therefore excluded from the analysis.

d). Disease progression. Further analysis of clinical records, radiology and biochemical screens is required before this can be reported.

e). Liver function. Further analysis of clinical records, radiology and biochemical screens is required before any potential effect of cannabinoids on liver function can be reported.

6. Overall bias.

Cannabis and placebo oil were developed by Little Green Pharma Ltd to be identical in taste, smell and appearance. It was provided to participants in matched opaque bottles, labelled to prevent any indication of allocated arm.

Cannabis and placebo oil were provided free of charge by Little Green Pharma Ltd who had no role in the design of the study, its execution, the collection or analysis of data, nor in the publication of results.
