# Supplementary material for: Medicinal cannabis for symptom control in advanced cancer: a double-blind, placebo-controlled, randomised clinical trial of 1:1 tetrahydrocannabinol and cannabidiol
Source: Support Care Cancer. 2025 Jul 24;33(8):715. doi: 10.1007/s00520-025-09763-5 (PMC12289739; doi:10.1007/s00520-025-09763-5)
Supplement: Supplementary file 2 — Supp Table 1 (DOCX 20.7 KB) [file 520_2025_9763_MOESM2_ESM.docx]

Supplementary Table 1. Dose schedule

|  |  | **THC/CBD**  **1:1 daily dose (10mg/10mg per mL)** | **mL/dose** | **mL/day** |
| --- | --- | --- | --- | --- |
| 0, 1 |  | 2.5mg/2.5mg | 0.25mL | 0.25mL |
| 2, 3 |  | 5mg/5mg | 0.5mL | 0.5mL |
| 4, 5 |  | 10mg/10mg | 0.5mL BD | 1mL |
| 6, 7 |  | 15mg/15mg | 0.5mL TDS | 1.5mL |
| 8, 9 |  | 20mg/20mg | 0.5mL  0.5mL  1mL | 2mL |
| 10, 11 |  | 25mg/25mg | 1mL  0.5mL  1mL | 2.5mL |
| 12, 13 |  | 30mg/30mg | 1mL TDS | 3mL |
| 14-28 |  | Continue final dose if perceived to be of benefit and tolerated | | |

THC/CBD, tetrahydrocannabinol/cannabidiol; BD, twice daily; TDS, three times daily
