# Supplementary material for: Medicinal cannabis for symptom control in advanced cancer: a double-blind, placebo-controlled, randomised clinical trial of 1:1 tetrahydrocannabinol and cannabidiol
Source: Support Care Cancer. 2025 Jul 24;33(8):715. doi: 10.1007/s00520-025-09763-5 (PMC12289739; doi:10.1007/s00520-025-09763-5)
Supplement: Supplementary file 3 — Supp Table 2 (DOCX 21.4 KB) [file 520_2025_9763_MOESM3_ESM.docx]

Supplementary Table 2. Opioid dose at days 14 and 28 (OME/24 hours, median (range))*

| **OME/24hrs** | **Baseline** | **Day 14** | **Day 28** |
| --- | --- | --- | --- |
| THC/CBD | 77 (0-520) | 80 (0-460) | 80 (0-530) |
| Placebo | 60 (0-2210) | 60 (0-2110) | 60 (0-1810) |

*includes patients who were started on opioids subsequent to baseline
