# Supplementary material for: Medicinal cannabis for symptom control in advanced cancer: a double-blind, placebo-controlled, randomised clinical trial of 1:1 tetrahydrocannabinol and cannabidiol
Source: Support Care Cancer. 2025 Jul 24;33(8):715. doi: 10.1007/s00520-025-09763-5 (PMC12289739; doi:10.1007/s00520-025-09763-5)
Supplement: Supplementary file 4 — Supp Table 3 (DOCX 22.9 KB) [file 520_2025_9763_MOESM4_ESM.docx]

Supplementary Table 3. EORTC QoL scores at baseline, day 14 and day 28

|  | **THC/CBD** | | | | | | **Placebo** | | | | | |
| --- | --- | --- | --- | --- | --- | --- | --- | --- | --- | --- | --- | --- |
|  | **Baseline** | | **Day 14** | | **Day 28** | | **Baseline** | | **Day 14** | | **Day 28** | |
| **Domain** | **N** | **Mean (SD)** | **N** | **Mean (SD)** | **N** | **Mean (SD)** | **N** | **Mean (SD)** | **N** | **Mean (SD)** | **N** | **Mean (SD)** |
| Physical functioning | 71 | 50.2 (26.7) | 53 | 51.3 (24.6) | 33 | 60.2 (25.6) | 72 | 54.6 (23.6) | 64 | 53.0 (25.9) | 50 | 53.5 (24.3) |
| Emotional functioning | 71 | 73.5 (23.4) | 53 | 80.0 (17.9) | 33 | 78.8 (17.8) | 72 | 73.3 (24.9) | 64 | 78.5 (23.6) | 50 | 78.8 (20.6) |
| Quality of life | 71 | 50.2 (23.3) | 53 | 57.2 (23.2) | 33 | 59.1 (16.7) | 71 | 46.9 (22.6) | 64 | 56.0 (26.0) | 50 | 56.7 (22.8) |
| Fatigue | 71 | 60.3 (24.5) | 53 | 57.2 (24.3) | 33 | 50.8 (24.5) | 72 | 56.8 (23.1) | 64 | 55.7 (25.2) | 50 | 54.4 (25.9) |
| Nausea and vomiting | 71 | 23.5 (32.8) | 53 | 17.6 (26.8) | 33 | 21.2 (24.0) | 71 | 21.1 (29.7) | 64 | 15.6 (23.9) | 50 | 11.3 (18.3) |
| Pain | 71 | 51.4 (32.8) | 53 | 33.0 (25.0) | 32 | 33.9 (24.5) | 71 | 46.2 (30.5) | 64 | 41.4 (28.8) | 50 | 39.0 (30.4) |
| Shortness of breath | 71 | 34.3 (28.7) | 53 | 35.2 (31.0) | 33 | 30.3 (31.6) | 72 | 35.6 (31.8) | 64 | 30.7 (31.0) | 50 | 24.7 (27.6) |
| Sleeplessness | 71 | 45.1 (33.9) | 53 | 27.0 (31.4) | 33 | 26.3 (26.0) | 72 | 47.2 (33.0) | 64 | 34.4 (34.1) | 50 | 34.0 (35.3) |
| Appetite loss | 71 | 48.8 (34.7) | 53 | 36.5 (32.2) | 33 | 35.4 (30.0) | 72 | 45.8 (37.3) | 64 | 37.0 (39.0) | 50 | 30.7 (31.5) |
| Constipation | 71 | 27.2 (32.0) | 53 | 22.0 (29.9) | 33 | 25.3 (30.1) | 72 | 22.7 (32.1) | 64 | 22.9 (26.5) | 49 | 23.1 (31.3) |

EORTC: European Organisation for Research and Treatment of Cancer, QoL: quality of life
