# Supplementary material for: Medicinal cannabis for symptom control in advanced cancer: a double-blind, placebo-controlled, randomised clinical trial of 1:1 tetrahydrocannabinol and cannabidiol
Source: Support Care Cancer. 2025 Jul 24;33(8):715. doi: 10.1007/s00520-025-09763-5 (PMC12289739; doi:10.1007/s00520-025-09763-5)
Supplement: Supplementary file 5 — Supp Table 4 (DOCX 21.1 KB) [file 520_2025_9763_MOESM5_ESM.docx]

Supplementary Table 4. Change in Depression, Anxiety and Stress Scores (DASS) from baseline to Day 14 and Day 28 (mean, SE)

| **Item, Timepoint** | **THC/CBD**  **Baseline to Day 14**  **n = 52** | **Placebo**  **Baseline to Day 14**  **n = 62** | **p-value*** | **THC/CBD**  **Baseline to Day 28**  **n = 33** | **Placebo**  **day Baseline to Day 28**  **n = 50** | **p-value*** |
| --- | --- | --- | --- | --- | --- | --- |
| DASS depression | -1.37 (0.34) | -1.15 (0.31) | 0.63 | -1.65 (0.44) | -1.09 (0.36) | 0.33 |
| DASS anxiety | -0.93 (0.35) | -1.05 (0.32) | 0.81 | -0.63 (0.51) | -1.4 (0.42) | 0.24 |
| DASS stress | -2.25 (0.43) | -1.95 (0.40) | 0.61 | -2.28 (0.54) | -2.31 (0.44) | 0.96 |

THC/CBD, Delta-9-Tetrahydrocannabinol/ Cannabidiol; DASS, Depression Anxiety Stress Scale; SE, standard error.

baseline adjusted p-values
