# Supplementary material for: Medicinal cannabis for symptom control in advanced cancer: a double-blind, placebo-controlled, randomised clinical trial of 1:1 tetrahydrocannabinol and cannabidiol
Source: Support Care Cancer. 2025 Jul 24;33(8):715. doi: 10.1007/s00520-025-09763-5 (PMC12289739; doi:10.1007/s00520-025-09763-5)
Supplement: Supplementary file 6 — Supp Table 5 (DOCX 29.4 KB) [file 520_2025_9763_MOESM6_ESM.docx]

Supplementary Table 5. Other adverse events

| **Other adverse event (N %)** | **THC/CBD**  N = 70* | **Placebo**  N = 72 | **p-value** |
| --- | --- | --- | --- |
| Paresthesia |  |  |  |
| No change/ better | 70 (100.0%) | 63 (87.5%) | 0.003 |
| New/ worse | 0 (0.0%) | 9 (12.5%) |  |
| Dysarthria |  |  |  |
| No change/ better | 68 (97.1%) | 71 (98.6%) | 0.62 |
| New/ worse | 2 (2.9%) | 1 (1.4%) |  |
| Lethargy |  |  |  |
| No change/ better | 66 (94.3%) | 72 (100.0%) | 0.06 |
| New/ worse | 4 (5.7%) | 0 (0.0%) |  |
| Peripheral sensory neuropathy |  |  |  |
| No change/ better | 68 (97.1%) | 67 (93.1%) | 0.44 |
| New/ worse | 2 (2.9%) | 5 (6.9%) |  |
| Tremor |  |  |  |
| No change/ better | 68 (97.1%) | 67 (93.1%) | 0.44 |
| New/ worse | 2 (2.9%) | 5 (6.9%) |  |
| Bloating |  |  |  |
| No change/ better | 70 (100.0%) | 71 (98.6%) | 1.00 |
| New/ worse | 0 (0.0%) | 1 (1.4%) |  |
| Ascites |  |  |  |
| No change/ better | 69 (98.6%) | 67 (93.1%) | 0.21 |
| New/ worse | 1 (1.4%) | 5 (6.9%) |  |
| GORD |  |  |  |
| No change/ better | 67 (95.7%) | 65 (90.3%) | 0.33 |
| New/ worse | 3 (4.3%) | 7 (9.7%) |  |
| Constipation |  |  |  |
| No change/ better | 56 (80.0%) | 59 (81.9%) | 0.77 |
| New/ worse | 14 (20.0%) | 13 (18.1%) |  |
| Oedema |  |  |  |
| No change/ better | 62 (88.6%) | 65 (90.3%) | 0.74 |
| New/ worse | 8 (11.4%) | 7 (9.7%) |  |
| Pain |  |  |  |
| No change/ better | 56 (80.0%) | 54 (75.0%) | 0.48 |
| New/ worse | 14 (20.0%) | 18 (25.0%) |  |
| Fatigue |  |  |  |
| No change/ better | 49 (70.0%) | 59 (81.9%) | 0.095 |
| New/ worse | 21 (30.0%) | 13 (18.1%) |  |

| Rash - maculo papular |  |  |  |
| --- | --- | --- | --- |
| No change/ better | 69 (98.6%) | 70 (97.2%) | 1.00 |
| New/ worse | 1 (1.4%) | 2 (2.8%) |  |
| Skin injury |  |  |  |
| No change/ better | 68 (97.1%) | 72 (100.0%) | 0.24 |
| New/ worse | 2 (2.9%) | 0 (0.0%) |  |
| Skin ulceration |  |  |  |
| No change/ better | 68 (97.1%) | 72 (100.0%) | 0.24 |
| New/ worse | 2 (2.9%) | 0 (0.0%) |  |
| Hot flashes |  |  |  |
| No change/ better | 69 (98.6%) | 70 (97.2%) | 1.00 |
| New/ worse | 1 (1.4%) | 2 (2.8%) |  |
| Thromboembolic event |  |  |  |
| No change/ better | 68 (97.1%) | 72 (100.0%) | 0.24 |
| New/ worse | 2 (2.9%) | 0 (0.0%) |  |
| Cough |  |  |  |
| No change/ better | 67 (95.7%) | 69 (95.8%) | 1.00 |
| New/ worse | 3 (4.3%) | 3 (4.2%) |  |
| Dyspnea |  |  |  |
| No change/ better | 61 (87.1%) | 63 (87.5%) | 0.95 |
| New/ worse | 9 (12.9%) | 9 (12.5%) |  |
| Myalgia |  |  |  |
| No change/ better | 69 (98.6%) | 71 (98.6%) | 1.00 |
| New/ worse | 1 (1.4%) | 1 (1.4%) |  |
| Lung infection |  |  |  |
| No change/ better | 68 (97.1%) | 72 (100.0%) | 0.24 |
| New/ worse | 2 (2.9%) | 0 (0.0%) |  |
| Depression |  |  |  |
| No change/ better | 66 (94.3%) | 69 (95.8%) | 0.72 |
| New/ worse | 4 (5.7%) | 3 (4.2%) |  |
| Anorexia |  |  |  |
| No change/ better | 65 (92.9%) | 65 (90.3%) | 0.58 |
| New/ worse | 5 (7.1%) | 1. (9.7%) |  |

*Two patients withdrew prior to day 2
