# Supplementary figures and images for: Medicinal cannabis for symptom control in advanced cancer: a double-blind, placebo-controlled, randomised clinical trial of 1:1 tetrahydrocannabinol and cannabidiol
Source: Support Care Cancer. 2025 Jul 24;33(8):715. doi: 10.1007/s00520-025-09763-5 (PMC12289739; doi:10.1007/s00520-025-09763-5)

Total OME (mg/24hr) at day 14

2000  
1500  
1000  
500  
0

THC/CBD:  $\rho = 0.01$ ,  $p = 0.91$

Placebo:  $\rho = -0.19$ ,  $p = 0.13$

1

2

3

Maximum dose of oil (ml)

Group    ● Placebo    ● THC.CBD

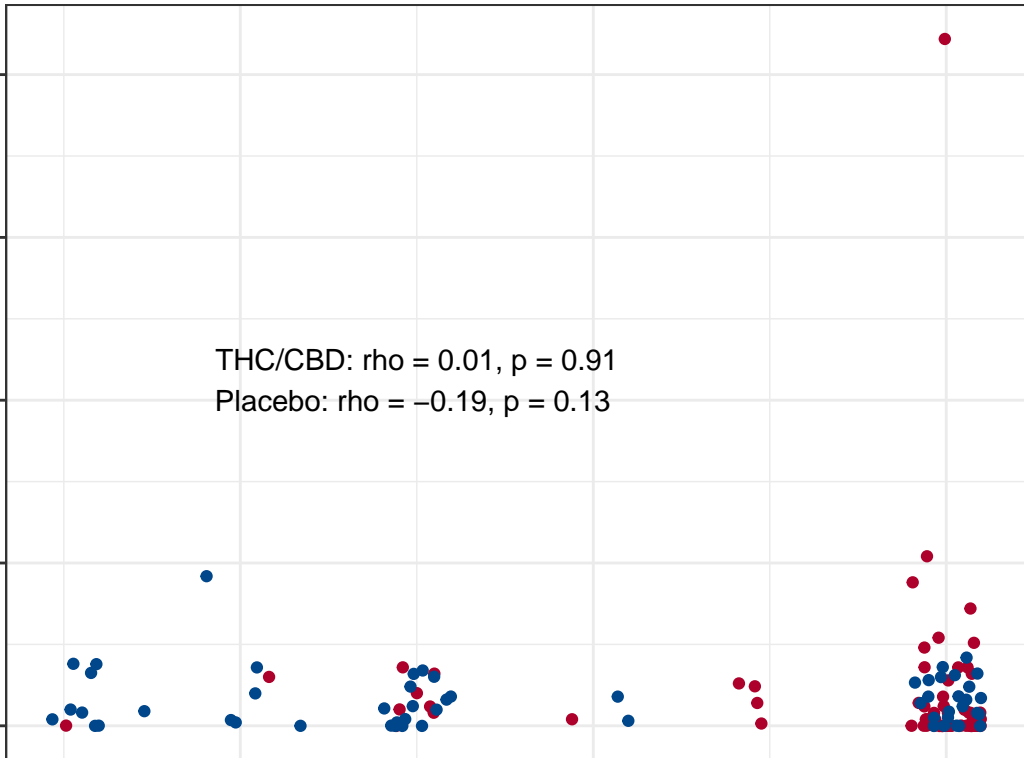

Supplement: Supplementary file 7 — Supp Figure 1. MC vs OME Scatter plot of maximum dose of oil vs total opioids (OME) at day 14 (PDF 16.4 KB) [file 520_2025_9763_MOESM7_ESM.pdf]

## Slide 1
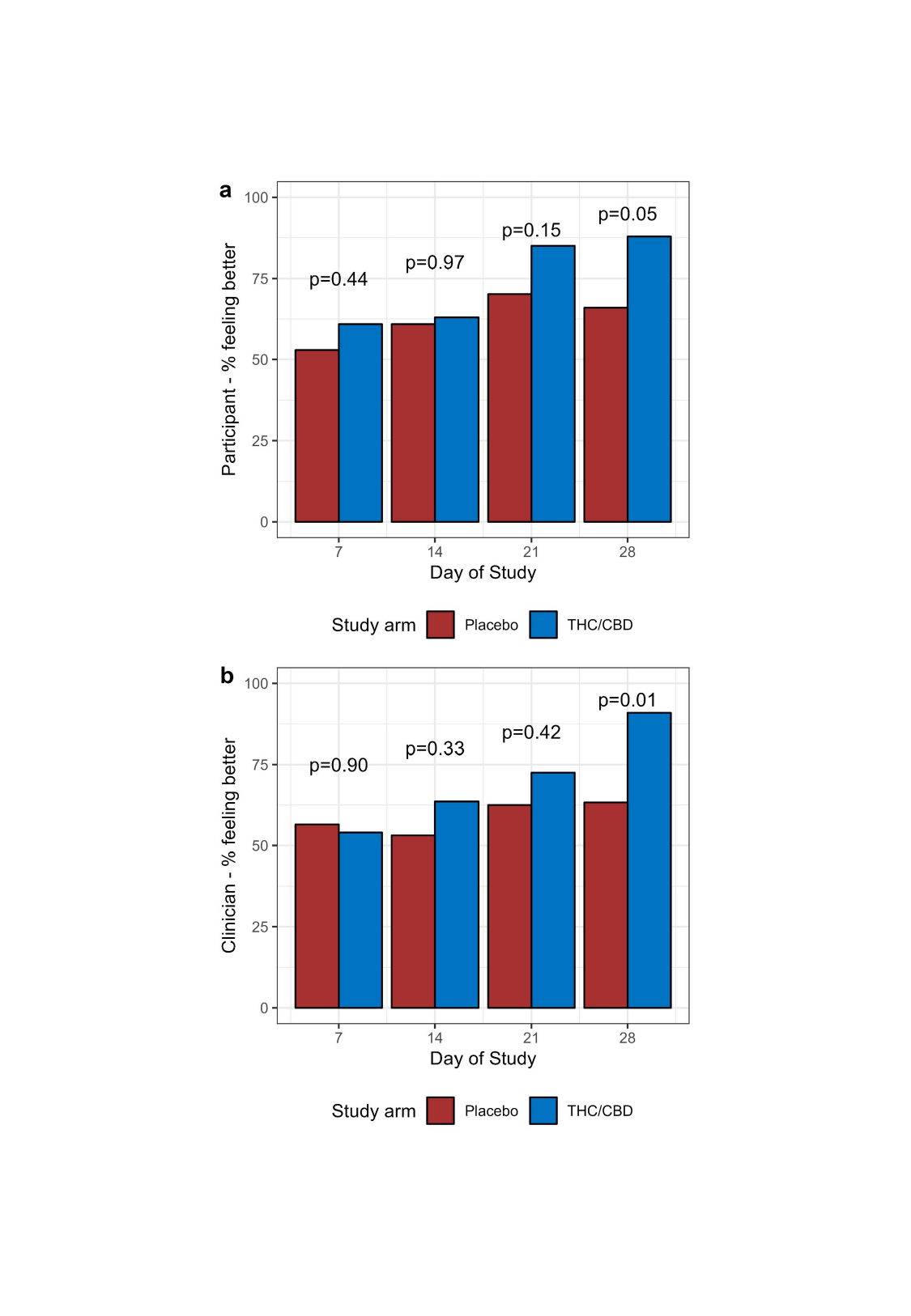

Supplement: Supplementary file 8 — Supp Figure 2. Global Impression of Change over time. a) patient assessed b) clinician assessed. THC/CBD, tetrahydrocannabinol/ cannabidiol (PPTX 694 KB) [file 520_2025_9763_MOESM8_ESM.pptx]

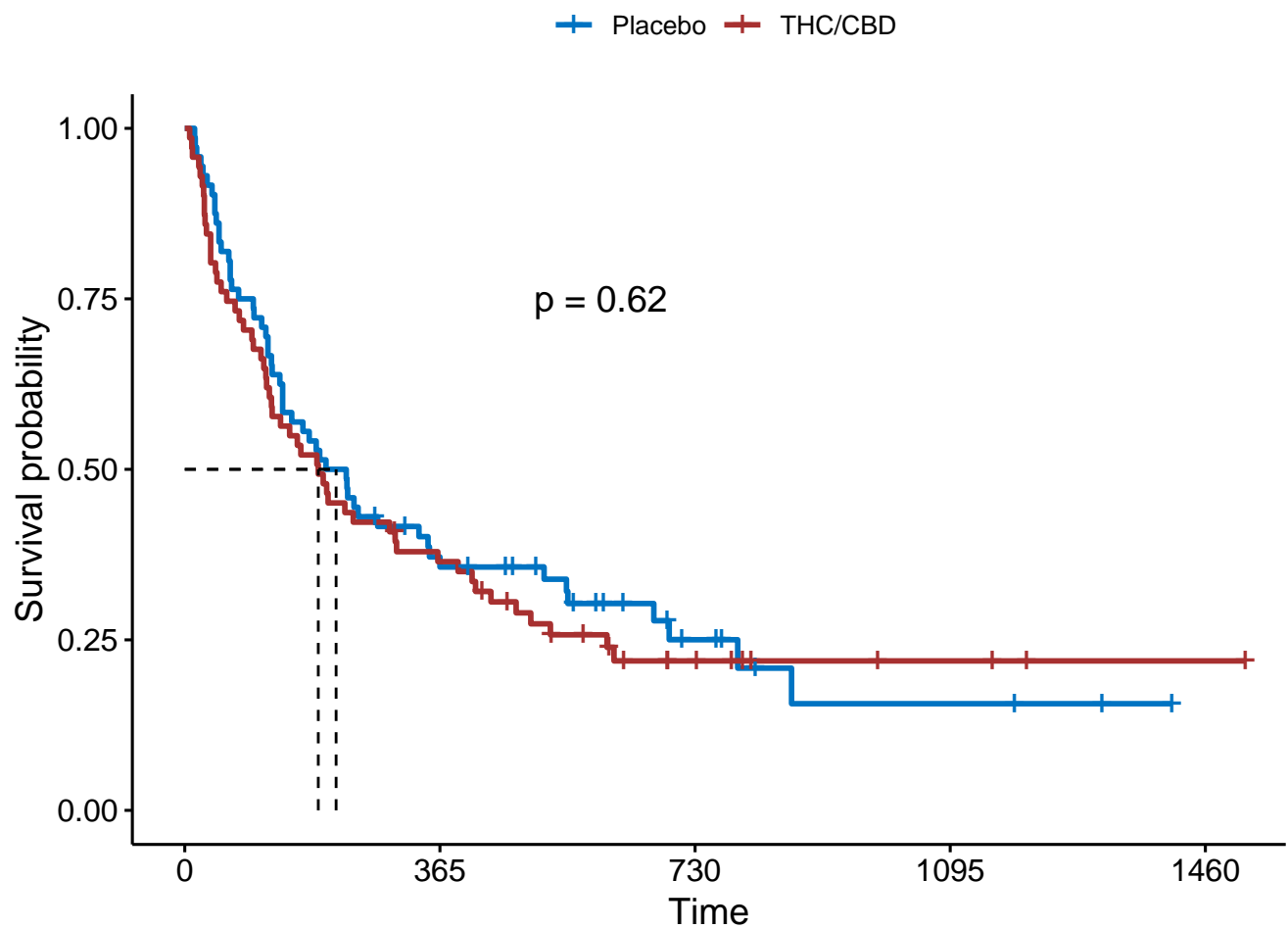

Number at risk

|         |    |    |   |   |   |
|---------|----|----|---|---|---|
| Placebo | 72 | 25 | 8 | 3 | 0 |
| THC/CBD | 71 | 25 | 8 | 3 | 1 |

Time

Supplement: Supplementary file 9 — Supp Figure 3. Survival over time. THC/CBD, tetrahydrocannabinol/ cannabidiol (PDF 8.42 KB) [file 520_2025_9763_MOESM9_ESM.pdf]
